# Transfusion‐transmitted malaria in India: A national survey of epidemiology and testing practices

**DOI:** 10.1111/vox.70261

**Published:** 2026-03-30

**Authors:** Jeremy W. Jacobs, Nabajyoti Choudhury, Satyam Arora, Aikaj Jindal, Nidhi Bhatnagar, Abhishekh Basavarajegowda, Shamee Shastry, Tulika Chandra, David Daniel, Steven J. Drews, Evan M. Bloch

**Affiliations:** ^1^ Department of Pathology, Microbiology and Immunology Vanderbilt University Nashville Tennessee USA; ^2^ Dibrugarh Cancer Centre Assam Cancer Care Foundation Dibrugarh India; ^3^ Department of Transfusion Medicine Post Graduate Institute of Child Health (PGICH) Noida Uttar Pradesh India; ^4^ Department of Transfusion Medicine Mohandai Oswal Hospital Ludhiana India; ^5^ Department of Immunohematology & Blood Transfusion B J Medical College Ahmedabad India; ^6^ Department of Transfusion Medicine Jawaharlal Institute of Postgraduate Medical Education & Research Puducherry India; ^7^ Department of Immunohematology and Blood Transfusion, Kasturba Medical College Manipal Academy of Higher Education Manipal Karnataka India; ^8^ Department of Transfusion Medicine King George Medical University Lucknow India; ^9^ Division of Transfusion Medicine, Department of Pathology Johns Hopkins University School of Medicine Baltimore Maryland USA; ^10^ Medical Microbiology & Surveillance and Discovery Laboratory, Donation Policy and Studies Canadian Blood Services Edmonton Alberta Canada; ^11^ Division of Diagnostic and Applied Microbiology, Department of Laboratory Medicine and Pathology University of Alberta Edmonton Alberta Canada

**Keywords:** blood donor, blood safety, haemovigilance, India, infectious disease, malaria, *Plasmodium*, rapid diagnostic test, transfusion‐transmitted infection

## Abstract

**Background and Objectives:**

Malaria still poses a public health burden in India despite ongoing elimination efforts. Blood donor screening for malaria is mandated in India, yet data on transfusion‐transmitted malaria (TTM) are lacking. We sought to evaluate testing practices for malaria, associated rates of positivity in donors and reported cases of TTM in India.

**Materials and Methods:**

We conducted a cross‐sectional survey of blood collection facilities in India from May to September 2025. Facilities were recruited through professional networks and direct outreach; responses were de‐identified. Data were collected on screening practices, malaria endemicity, quality control measures and TTM cases from 2020 to 2024.

**Results:**

Among 262 facilities, 256 (97.7%) reported routine malaria screening of all donations, most commonly using antigen rapid diagnostic tests (RDTs) (228/256, 89.1%). From 2020 to 2024, facilities tested 9,275,688 donations, with 1231 confirmed positive donations (overall 13.27 per 100,000 units) and a rising annual positivity rate from 3.4 to 22.0 per 100,000 units. Only eight facilities (3.1%) reported TTM cases, and less than half of facilities (116/256, 45.3%) participated in external quality assessment (EQA) and/or performed lookback investigations (88/262, 33.6%).

**Conclusion:**

In this national survey sample in India, almost all facilities reported routine malaria screening of blood donations, mostly using RDT. The findings suggest that rates of donor positivity may have increased, yet TTM is under‐recognized and/or unreported. Strengthening EQA and haemovigilance, particularly in malaria endemic regions, is needed to safeguard the blood supply, particularly as India strives towards malaria elimination.

**Highlights**

- Near‐universal malaria screening is in place across surveyed Indian blood centres, dominated by antigen rapid diagnostic tests.
- Although the reported transfusion‐transmitted malaria is rare, *Plasmodium* reactivity in donors increased substantially from 2020 to 2024.
- A major vulnerability is quality systems and haemovigilance, as less than half of facilities participated in external quality assessment and only one‐third performed lookback investigations.

## INTRODUCTION

Malaria remains a significant public health challenge in India. In 2024, the country reported approximately 2 million cases of malaria [1, 2]. India accounts for 0.7% of all malaria cases and 0.6% of global malaria deaths, representing approximately three‐quarters of all malaria cases in the World Health Organization (WHO) South‐East Asia Region [2]. Multiple states and union territories are impacted by ongoing transmission, yet many districts have achieved zero cases [1]. *Plasmodium vivax* and *Plasmodium falciparum* are the dominant species in India, with their relative proportions varying by region. Almost half of the global burden of malaria due to *P. vivax* is in India [2].

Transfusion‐transmitted malaria (TTM) poses a threat to blood safety, as asymptomatic parasitaemia in blood donors can persist for months or years [3, 4, 5]. There are no pathogen reduction technologies for red blood cells or whole blood currently in clinical use (i.e., outside of research settings) [6]. *Plasmodium* parasites can survive storage in refrigerated blood components for days to weeks, potentially remaining viable for transfusion‐transmission [7, 8, 9]. The risk is particularly concerning in malaria endemic regions where the prevalence of asymptomatic parasitaemia among donors may be high, yet transfusion recipients may have limited prior immunity (e.g., due to young age) or have compromised immune systems due to underlying disease, pregnancy or senescence [10].

At the time of writing, India mandates universal malaria screening for all blood donations under the Drugs and Cosmetics Rules [11, 12]. The second edition of the standards from the National Blood Transfusion Council (NBTC), published in 2022, recommends that blood centres use a validated and sensitive antigen test [13]. Individuals with a history of malaria are eligible to donate 3 months after full recovery and completion of treatment, provided they are asymptomatic and meet all other donor eligibility criteria [11, 13]. In practice, this is operationalized through pre‐donation history/medical assessment together with routine malaria screening of the donation; however, there is no clear and standardized national guidance on how blood centres should independently verify prior infection, treatment completion, recovery or asymptomatic status at donor re‐entry. Despite these policies, the effectiveness of blood donor screening, the burden of TTM and the variation in testing methods across Indian blood collection facilities remain poorly characterized. Prior studies have largely been confined to single centres or specific geographic regions, and reviews of TTM in the Indian subcontinent suggest that extant data are incomplete [14, 15]. Accordingly, we sought to evaluate contemporary transfusion screening practices for malaria in India, with special attention to the reported rates of TTM.

## MATERIALS AND METHODS

### Study design and setting

India has a large, heterogeneous blood transfusion system comprising more than 3800 licensed blood centres that collect about 14 million units of blood annually; most donations are from voluntary, non‐remunerated blood donors, but there is still ongoing reliance on family/replacement donors in some regions [16, 17]. Within this system, we conducted a cross‐sectional survey of blood collection facilities across India from May to September 2025. The survey was developed in research electronic data capture [18, 19] hosted at Johns Hopkins University and distributed electronically through professional networks and direct outreach to facilities by study investigators and collaborators in India. Eligible respondents included physicians, transfusion medicine specialists, blood centre directors, laboratory supervisors and other personnel knowledgeable about malaria screening and haemovigilance practices at their facility. Participation was voluntary, and responses were de‐identified. Respondents were asked to provide the name of their facility to prevent duplicate responses from being included in the analysis. Response quality was assessed by screening for duplicate institutional submissions, incomplete questionnaires and responses lacking permission for research use; such responses were excluded from the analysis. Because the survey was distributed through overlapping professional channels and direct forwarding, the total number of facilities reached could not be precisely determined. This study was approved by the Johns Hopkins University Institutional Review Board (IRB00482730). Because the project involved a voluntary, de‐identified survey of institutions and did not include patient enrolment, clinical intervention or collection of identifiable donor‐ or patient‐level data, no additional Indian regulatory authorization was sought.

### Data collection

The structured questionnaire (Supporting Information) captured information on (1) facility characteristics including organization type, location and donation volumes; (2) malaria endemicity classification (self‐reported); (3) testing methodologies and nucleic acid testing (NAT) capabilities; (4) quality control measures including external quality assessment (EQA), repeat testing protocols and confirmatory testing practices; (5) haemovigilance participation; (6) TTM case reporting (TTM was defined by each responding facility) with details on blood products implicated and patient populations affected and (7) annual testing volumes and positivity rates from 2020 to 2024. Testing methods were categorized as light microscopy, rapid diagnostic tests (RDTs), formal laboratory tests (automated/semi‐automated immunoassays), molecular testing (nucleic acid amplification) or antibody testing. For the purposes of this study, the terms ‘molecular testing’ and ‘NAT were used interchangeably.

### Operational definitions

Because this was a survey‐based study, responses were analysed as reported by each participating facility. Facility endemicity status, malaria testing practices, confirmed positive donations and reports of TTM were based on respondent report according to local facility practices and definitions; these were not independently verified or centrally adjudicated by the study team.

### Statistical analysis

Descriptive statistics were reported as frequencies and proportions for categorical variables. The primary outcome was the annual malaria screening positivity rate, defined as confirmed positive units divided by total units screened per facility per year, expressed per 100,000 units, with 95% confidence intervals (CIs) derived using the Poisson exact (Garwood) method. Facilities reporting zero units screened in a given year were excluded from that year's rate calculations.

Temporal trends in positivity rates across 2020–2024 were assessed using two methods. The Cochran–Armitage trend test evaluated trends in the proportion of positive units over ordered annual time points, accounting for variation in testing volumes across years. A log‐linear regression model was additionally fitted, with calendar year as the predictor and the natural log of total units screened as an offset, to estimate the annual incidence rate ratio (IRR) with 95% CI. Both analyses were repeated within self‐reported endemicity subgroups (endemic and non‐endemic). Annual positivity rates were compared between endemic and non‐endemic facilities using rate ratios (RRs) with 95% CIs, calculated using a log‐normal approximation. Comparisons of categorical variables between facility groups were performed using Pearson's chi‐square test of independence; Fisher's exact test was substituted when any expected cell count was fewer than five. All tests were two‐sided, with statistical significance set at *p* < 0.05. Given the exploratory nature of the survey, no adjustment for multiple comparisons was applied. Descriptive analyses and chi‐square tests were performed using Prism version 10.6.1 (GraphPad Software, La Jolla, CA, USA); temporal trend analyses were performed using Python 3.12.3 with the SciPy library (version 1.17.0; SciPy.org).

## RESULTS

### Facility characteristics

A total of 348 responses were received. Because dissemination occurred through overlapping professional channels and forwarding by recipients, the exact number of facilities that received the questionnaire could not be determined, precluding formal response rate calculation. Among these, 33 facilities had more than one respondent (*n* = 72 responses), and therefore they were excluded from the analysis. Of the remaining 276 responses, 14 respondents either did not complete the survey or did not grant permission to use their answers for research purposes. Therefore, the final analysis comprised 262 facilities (Table 1). The majority were private institutions (*n* = 130, 49.6%) or non‐governmental/charitable organizations (*n* = 69, 26.3%). Facilities were distributed across multiple states (24 of the 36 states/union territories in India), with the highest representation from Haryana, Punjab, Gujarat and Maharashtra (Table S1). Regarding donation volumes in 2024, most facilities collected 1000–4999 (*n* = 86, 32.8%) or 5000–9999 (*n* = 79, 30.2%) whole‐blood units.

**TABLE 1:** Distribution of facilities by organization type and whole‐blood donation volume in 2024 (*N* = 262).

Organization type	*N*	Percentage (%)
Private	130	49.6
Non‐governmental/charitable	69	26.3
Governmental/public	58	22.1
Other	5	1.9

Donation volume category	*N*	Percentage (%)
<1000 units	20	7.6
1000–4999 units	86	32.8
5000–9999 units	79	30.2
10,000–24,999 units	56	21.4
25,000–49,999 units	15	5.8
50,000–99,999 units	2	0.8
No response	4	1.5

### Malaria endemicity

Among the 262 facilities, 136 (51.9%) reported being located in a malaria‐endemic area, while 94 (35.9%) reported being located in a non‐endemic area. An additional 30 facilities (11.5%) were uncertain about their endemic classification, and 2 (0.8%) did not respond. Among the 136 respondents that reported being located in malaria‐endemic areas, 134 provided information on the species the most frequently identified locally. Of these, 39 (29.1%) reported *P. vivax* and 15 (11.2%) reported *P. falciparum* as the predominant species, whereas 80 (59.7%) were unsure of the predominant species in their area (Table S2).

### Malaria testing practices

Malaria screening was nearly universal, with 256 of 262 facilities (97.7%) performing routine testing on all donations; only 4 facilities (1.5%) did not test routinely, while 2 (0.8%) did not respond (Table 2). Among the 256 facilities performing testing, antigen RDTs were the predominant screening method, used by 228 facilities (89.1%). Other methods included light microscopy (23 facilities, 9.0%), formal laboratory immunoassays (21 facilities, 8.2%), antibody tests (7 facilities, 2.7%) and molecular testing (7 facilities, 2.7%). The latter was concentrated in Northern (*n* = 4; Uttar Pradesh, Delhi and Rajasthan), Central (*n* = 2; Chhattisgarh and Madhya Pradesh) and Western (*n* = 1; Gujarat) regions, with no NAT‐based malaria screening reported from the Southern, Eastern or Northeastern regions. Three facilities (1.2%) did not specify their testing methodology. After a reactive screening result, repeat testing with the same method was reported by 117 facilities (45.7%), while confirmatory testing with an alternative method was performed by 95 (37.1%).

**TABLE 2:** Malaria screening practices.

Routine malaria testing		
Category	*N*	Percentage (%)
Perform routine testing on all donations	256	97.7
Do not routinely test	4	1.5
No response	2	0.8

Testing methods (among 256 facilities performing testing)		
Method	*N*	Percentage (%)
Antigen rapid diagnostic tests (RDTs)	228	89.1
Light microscopy	23	9.0
Formal laboratory immunoassays	21	8.2
Antibody tests	7	2.7
Molecular testing	7	2.7
Did not specify testing methodology	3	1.2

Species identification		
Practice	*N*	Percentage (%)
Do not identify malaria species	163	63.7
Only differentiate *Plasmodium falciparum* from non‐*P. falciparum*	45	17.6
Perform full species identification	43	16.8
No response	5	2.0

*Note*: Percentages for routine testing calculated from total *N* = 262 facilities. Testing method percentages calculated from *N* = 256 facilities that perform testing. Species identification percentages calculated from total *N* = 256 facilities performing testing. Multiple testing methods could be used by individual facilities.

Regarding species identification testing practices, 163 facilities (63.7%) did not identify malaria species, 45 (17.6%) only attempted to differentiate *P. falciparum* from non‐*P. falciparum* species and 43 (16.8%) attempted full species identification. Five facilities did not respond.

While only 7 facilities performed NAT for malaria, NAT was available at 78 facilities (29.8%) for human immunodeficiency virus (HIV), hepatitis B virus (HBV) and hepatitis C virus (HCV). The majority of facilities (184, 70.2%) did not have NAT capabilities for any infectious disease marker.

Quality control measures for malaria screening showed considerable variation across facilities. Among the 256 facilities that reported testing for malaria, EQA participation was reported by 116 facilities (45.3%), while 130 (50.8%) did not participate, 8 (3.1%) were uncertain and 2 (0.8%) did not respond.

**FIGURE 1:**
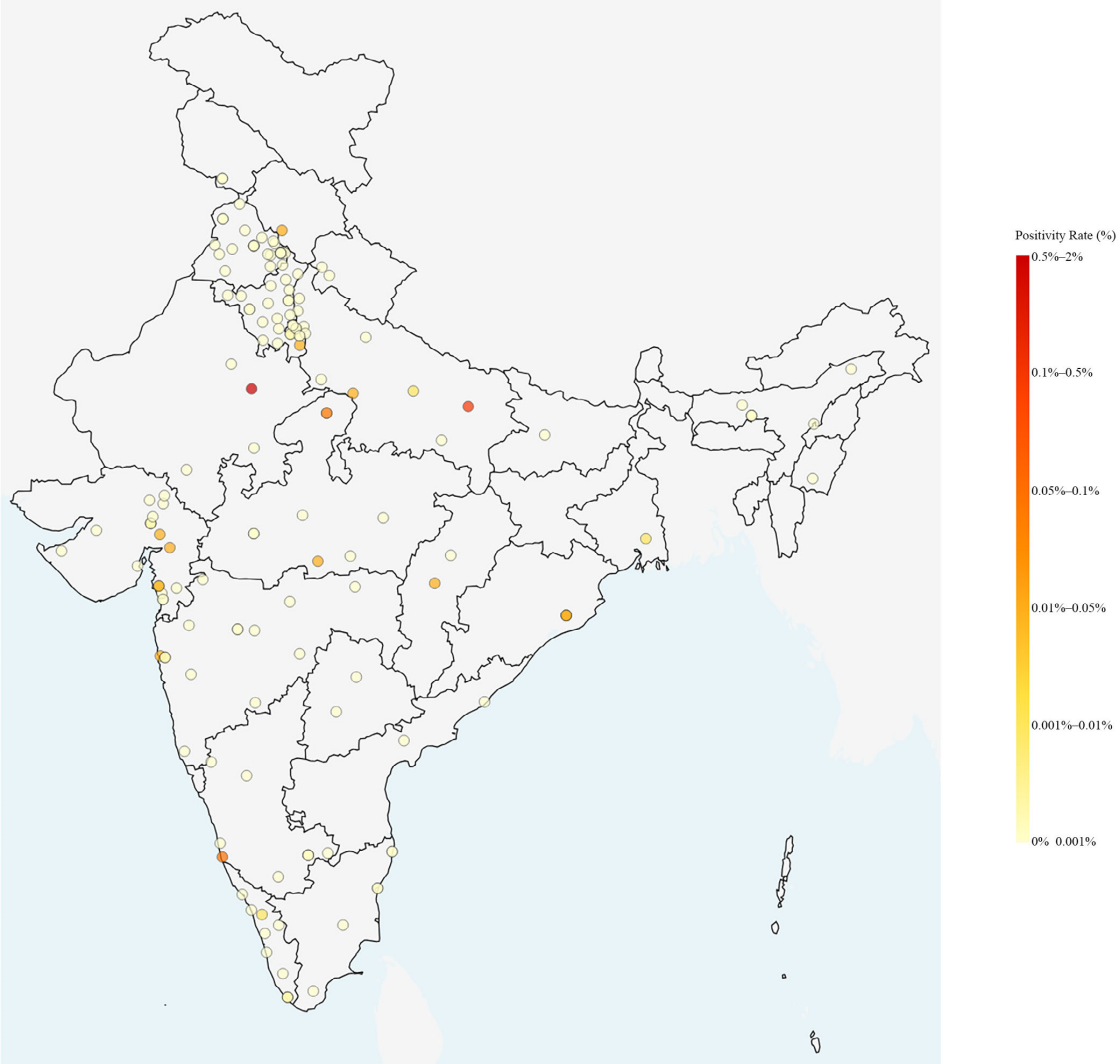
Map of survey respondents and reported *Plasmodium* positivity rates for donated units. [Correction added on 7 April 2026, after first online publication: Figure 1 was corrected.]

Over the 5‐year period from 2020 to 2024, participating facilities tested a cumulative total of 9,275,688 units for malaria, of which 1231 were confirmed positive for malaria, yielding an overall positivity rate of 13.27 per 100,000 units (Table 3, Figure 1).

**TABLE 3:** Annual testing volumes and malaria positivity rates (2020–2024).

Year	Facilities reporting	Total units tested	Total positive tests (no. facilities with at least 1 positive)	Rate per 100,000 units
2020	217	1,570,779	54 (*n* = 16)	3.44
2021	218	1,721,064	59 (*n* = 16)	3.43
2022	222	1,887,821	299 (*n* = 19)	15.84
2023	231	2,006,333	360 (*n* = 17)	17.94
2024	238	2,089,691	459 (*n* = 22)	21.96
Total	–	9,275,688	1231	13.27

*Note*: Reporting facilities are those that reported at least 1 unit of blood donated during the specific year.

### TTM cases

Only 8 of the 262 facilities (3.1%) reported cases of TTM during the 2020–2024 period; 236 (90.1%) had not encountered any cases, 15 (5.7%) were uncertain and 3 (1.1%) did not respond. Four of the eight facilities were located in the west (Maharashtra, *n* = 3; Gujarat, *n* = 1), two were in the south (Andhra Pradesh, *n* = 1; Tamil Nadu, *n* = 1) and two were in the north (Uttar Pradesh, *n* = 2). Among the eight facilities that reported cases of TTM, three facilities provided specific counts totalling four cases; one facility reported ‘fewer than 50 cases’ without a precise number; and four facilities indicated at least one TTM case but did not quantify the total. Thus, there were a minimum of nine cases identified, although the total number of cases is unknown since not all of the facilities that reported encountering at least one case provided specific case numbers. Among facilities reporting TTM cases, seven implicated whole blood, three implicated red cells and two implicated platelets. Regarding the affected patient populations, immunocompromised patients were identified in three cases (37.5%), while no TTM cases were reported in pregnant patients or children among the surveyed facilities.

### Haemovigilance and investigation practices

Haemovigilance reporting was done by 130 of 262 facilities (49.6%), while 124 (47.3%) did not report to any haemovigilance programme and 8 (3.1%) did not respond (Table S3). Lookback investigations following identification of an infected donation were conducted by 88 facilities (33.6%), with 163 (62.2%) not performing lookback and 11 (4.2%) not responding. Among facilities conducting lookback, the investigated time period varied: 23 facilities (27.4%) investigated up to 3 months, 20 (22.7%) investigated from 3 to 6 months, 14 (15.9%) investigated from 6 to 12 months, 13 (14.8%) investigated beyond 12 months from the index donation, 14 (15.9%) were not sure and 4 (4.5%) did not respond.

### Comparison of endemic and non‐endemic areas

Routine malaria testing practices were similar between facilities in endemic (*n* = 136) and non‐endemic (*n* = 94) regions; 97.1% and 97.9% performed routine malaria testing, respectively. However, EQA participation was higher in non‐endemic compared to endemic facilities (61/94 [64.9%] vs. 39/136 [28.7%]; *p* < 0.001). TTM cases were more commonly reported in non‐endemic facilities (5/94, 5.3%) compared to endemic facilities (3/136, 2.2%), although the absolute numbers remained low in both groups and the difference was not statistically significant (odds ratio 2.49, 95% CI: 0.58–10.69; *p* = 0.28). Facilities in the endemic regions had a higher rate of positive units compared to those in non‐endemic regions (3.59 per 100,000 [95% CI: 3.10–4.15 per 100,000] vs. 2.68 per 100,000 [95% CI: 2.20–3.28 per 100,000]; *p* = 0.02) (Table 4).

**TABLE 4:** Annual testing volumes and malaria positivity rates (2020–2024) between facilities in endemic regions and non‐endemic regions.

Year	Facilities reporting		Total units tested		Total positive tests (no. facilities with ≥1 positive)		Rate per 100,000 units (95% CI)		Rate ratio (95% CI)	*p*‐value
	Non‐endemic	Endemic	Non‐endemic	Endemic	Non‐endemic	Endemic	Non‐endemic	Endemic		
2020	79	119	595,443	878,624	15 (*n* = 3)	26 (*n* = 11)	2.52 (1.41–4.15)	2.96 (1.93–4.34)	1.17 (0.62–2.22)	0.62
2021	80	119	670,245	945,934	18 (*n* = 5)	26 (*n* = 10)	2.69 (1.59–4.24)	2.75 (1.80–4.03)	1.02 (0.56–1.87)	0.94
2022	80	120	736,702	1,025,984	16 (*n* = 7)	27 (*n* = 10)	2.17 (1.24–3.53)	2.63 (1.73–3.83)	1.21 (0.65–2.25)	0.54
2023	84	123	784,073	1,083,797	23 (*n* = 6)	47 (*n* = 8)	2.93 (1.86–4.40)	4.34 (3.19–5.77)	1.48 (0.90–2.43)	0.12
2024	86	124	826,725	1,108,754	25 (*n* = 9)	55 (*n* = 10)	3.02 (1.96–4.46)	4.96 (3.74–6.46)	1.64 (1.02–2.63)	0.04
Total	–	–	3,613,188	5,043,093	97	181	2.68 (2.20–3.28)	3.59 (3.10–4.15)	1.34 (1.04–1.71)	0.02

*Note*: Facilities reporting uncertainty about endemicity status (*n* = 30) and non‐respondents (*n* = 2) were excluded.

Abbreviation: CI, confidence interval.

## DISCUSSION

This national survey of a convenience sample of 262 Indian blood collection facilities affords insight into contemporary malaria screening practices and the epidemiology of TTM in India. The major findings include the near‐universal adoption of malaria screening of blood donations with predominant use of RDTs, with a more than four‐fold increase in donor malaria positivity rates observed from 2020 to 2024. Despite the observed increase in detected positive donor units, there were very few reported cases of TTM. However, the low numbers of reported cases need to be considered in the context of suboptimal EQA and haemovigilance, particularly in endemic areas. These findings highlight both the successful implementation of blood donor screening for malaria in India, as well as the vulnerabilities in the blood system with opportunities for improvement.

Our results extend prior work on TTM in the Indian subcontinent, which has largely relied on single‐centre series or regional data [20, 21, 22]. A 2025 systematic review synthesized evidence from 122 studies and more than 6.5 million individuals, reporting a prevalence of donor parasitaemia of up to 0.87% by microscopy and 2.3% by RDT in the Indian subcontinent and neighbouring countries [14]. That review emphasized the absence of coordinated national data and variability in screening strategies. Our standardized facility‐level survey addresses this gap by providing, to our knowledge, one of the first national scale multi‐facility snapshots of malaria screening practices across India. Consistent with regulatory requirements for universal malaria testing [11], the large majority of responding facilities reported performing routine screening of all donations, predominantly using RDTs. However, as this study relied on self‐reported data without on‐site validation, these findings reflect reported rather than directly observed practices; the extent to which reported practices align with actual implementation may vary, and facilities with more established programmes may be over‐represented among respondents.

The reported malaria positivity rate among donors represents a four‐fold increase from 2020 to 2024. There are several possible explanations—real and artefactual—for the observed increase. These include the rigour of data collection during a particular year, changes in local transmission (i.e., ecological variation), expansion of testing to higher risk regions, improved uptake and performance of RDTs and/or shifts in the donor population (e.g., greater proportion of donors from higher transmission regions or those at higher risk of malaria infection). The observed temporal increase also overlaps with the COVID‐19 pandemic, during which interruptions in vector control and health services may have influenced malaria surveillance and reporting [23, 24]. Notably, single‐centre studies from India have reported stable or declining positivity rates over similar timeframes—a 9‐year retrospective study from Jharkhand documented a decline from 0.11% in 2014–2015 to 0.006% by 2022–2023 [21], and a study from Uttarakhand reported a positivity rate of 0.05% in 2021 [20]—suggesting that the aggregate upward trend observed in our multi‐facility survey may not be apparent from individual institutional data. This discordance may reflect geographic heterogeneity in malaria transmission, differences in facility catchment populations and the influence of a small number of high‐volume facilities on aggregate rates. Importantly, because positivity data were self‐reported retrospectively, the observed trend is also susceptible to variability in local data quality and record‐keeping practices across facilities and years, which should be considered when interpreting the magnitude of the increase. Nonetheless, given India's broader progress towards malaria elimination [1, 2], even low absolute donor positivity rates are important, whereby any infected donation may be sufficient to cause TTM in malaria‐native (non‐immune) and/or immunocompromised recipients [3, 4, 5, 7]. Our findings underscore the need for ongoing donor surveillance to complement routine population‐level malaria efforts.

The predominant use of RDT‐based screening is likely informed by cost and logistical considerations, such as rapid turnaround and ease of use [25]. All the testing modalities that are in use for clinical malaria have limitations in the context of blood donor screening. Microscopy is labour‐intensive and insensitive at low levels of parasitaemia, which is to be expected in blood donors who feel sufficiently well to donate. RDTs may have lower sensitivities if parasitaemia is low; they also risk false‐negative results in cases of *pfhrp2/3* gene deletions [25, 26, 27, 28, 29]. Like microscopy, RDTs are manual and labour‐intensive. Further, many RDTs only distinguish between *P. falciparum* and non‐*P. falciparum* results, where determination of species has implications for donor counselling and clinical management of TTM cases should they occur [25].

Automated approaches, such as serology and NAT, have a higher degree of complexity, requiring equipment, reagents and skilled personnel, which are therefore more expensive. Of note, less than a third of responding facilities in our survey reported the use of NAT for the major transfusion‐transmitted viruses (i.e., HIV, HBV and HCV) despite this being the standard in high‐income countries [30]. Less than 3% of facilities reported molecular testing for malaria, specifically. The role of NAT in malaria screening is an active area of debate in high‐income (non‐endemic) countries, as novel high‐throughput molecular assays for donor malaria screening have been developed [31].

The low reported incidence of TTM—only eight facilities indicated cases of TTM over 5 years—is not reassuring. Of these eight facilities, three self‐reported being located in malaria‐endemic areas, while five reported being in non‐endemic areas, illustrating that TTM continues to occur in both malaria endemic and non‐endemic regions despite stringent donor deferral policies and increasingly sensitive testing [3, 7, 32, 33]. The low reported numbers of TTM in our survey highlight the challenges around post‐transfusion surveillance, defining imputability to transfusion, and passive reporting rather than risk. Counterintuitively, a greater proportion of non‐endemic facilities reported TTM than endemic facilities. One possible explanation is that, in settings where malaria is rare, a case of TTM may be more readily recognized as unusual and investigated. By contrast, it is challenging to distinguish TTM from community‐acquired malaria in an endemic area, where there may be antecedent infection in donors and recipients alike. Another consideration is that this geographic distribution may partly reflect the broader sampling pattern of the survey, with under‐representation of high‐burden regions, and should be interpreted accordingly.

Our survey identifies gaps in quality control and haemovigilance that could blunt the effectiveness of screening [34, 35]. Less than half of facilities participated in EQA for malaria testing and repeated or confirmatory testing of reactive donations was not universal. Facilities in endemic regions were less likely than those in non‐endemic regions to engage in EQA, despite likely higher risk of malaria. Participation in formal haemovigilance programmes and lookback investigations after detecting infected donations was similarly incomplete. These findings suggest that the investment in test procurement is not matched by rigorous quality systems and surveillance [34].

This study has several limitations. Because survey invitations were distributed through professional networks and national organizations rather than through a registry of all facilities, it is unknown how many facilities received the survey; therefore, we could not calculate a formal response rate, and the sample may over‐represent larger or more engaged facilities. Similarly, the survey relied on self‐reported data and voluntary participation, which may have introduced selection bias favouring facilities with more established testing programmes. Small and/or more resource‐limited centres, including those in remote areas, may be under‐represented. We did not specify how TTM should be defined, nor did we ask how respondents defined it. The survey captured practices and cases reported through 2024, but actual TTM incidence may be underestimated due to underdiagnosis and under‐reporting. Retrospective data on testing volumes and positivity rates may be subject to recall bias or incomplete record keeping. We did not independently verify the sensitivity and specificity of testing platforms used by individual facilities, nor did we evaluate the residual risk of TTM (i.e., the probability that an infected blood donation gives a negative result on screening tests). An additional vulnerability is the limited clarity around re‐entry of donors with prior malaria. Although Indian guidance permits donation 3 months after full recovery, our survey did not assess how centres verify prior infection history, treatment completion or asymptomatic status at the time of return, or whether any centres required supplemental retesting beyond routine donation screening. This is consequential because previously infected but asymptomatic donors have been implicated in TTM, and routine screening methods, particularly RDT‐based approaches, may miss low‐level parasitaemia. Furthermore, our classification of endemic versus non‐endemic status relied on respondents' self‐report and may not perfectly align with national or WHO categorizations, and not all facilities were able to classify their own endemicity status. State‐level analysis was further constrained by sparse representation; 20 of the 24 states with responding facilities had fewer than 10 respondents, precluding reliable subnational inference. Notably, India's highest burden states for malaria, including Odisha, Chhattisgarh, Madhya Pradesh and states of the Northeast, were under‐represented, which may have caused our survey to underestimate the burden of TTM risk in the facilities and populations where it is likely greatest.

Despite these limitations, our findings have important implications for blood safety policy in India and other malaria‐endemic countries. First, donor testing for malaria must be coupled with strengthened EQA, particularly in endemic regions where donor positivity rates are highest. Second, haemovigilance systems should be reinforced to ensure standardized case definitions with imputability, routine reporting of suspected TTM and structured lookback investigations whenever malaria‐positive donations are identified [36]. In our survey, lookback intervals varied widely, from ≤3 months to >12 months, underscoring the need for clear national guidance on minimum lookback intervals and investigation procedures. Third, donor surveillance data from blood centres should be integrated into national malaria control programmes, using donor positivity as a sensitive signal for local transmission and for periodic re‐evaluation of testing algorithms. Embedding blood safety interventions within broader, multimodal infectious disease elimination efforts—through coordinated improvements in testing, data systems and regulation—can yield substantial collateral benefits for transfusion safety. In India, malaria donor screening may represent a practical and underused component of the broader malaria elimination agenda. Experiences from other settings illustrate how integrating blood safety measures within wider infectious disease control strategies can strengthen transfusion safety systems [37, 38, 39].

In conclusion, this national survey provides one of the first multi‐institutional assessments of malaria donor screening practices across India. Most responding facilities reported performing routine RDT‐based screening consistent with regulatory requirements, although these findings reflect reported rather than verified practices. Of particular concern, facilities in endemic regions reported weaker quality infrastructure than non‐endemic counterparts across multiple domains. Strengthening quality systems in endemic settings, aligning facility‐level endemicity classification with national surveillance data and integrating blood donor positivity as a sentinel epidemiological signal within India's malaria elimination programme are priorities that merit urgent attention. Blood safety and malaria control must advance in concert if India is to achieve its 2030 elimination goal.

## CONFLICT OF INTEREST STATEMENT

E.M.B. reports personal fees and non‐financial support from Terumo BCT, Grifols, Abbott Laboratories and UptoDate, outside of the submitted work. E.M.B. is a member of the US Food and Drug Administration (FDA) Blood Products Advisory Committee. Any views or opinions that are expressed in this report are those of the author's, based on his own scientific expertise and professional judgement; they do not necessarily represent the views of either the Blood Products Advisory Committee or the formal position of FDA, and do not bind or otherwise obligate or commit either Advisory Committee or the Agency to the views expressed.

## Supporting information


**Table S1.** Geographic distribution of included facilities in India (*N* = 262).
**Table S2**. Facility‐reported predominant *Plasmodium* species among facilities self‐reporting location in malaria‐endemic areas, stratified by state/union territory.
**Table S3**. Haemovigilance practices by region (*N* = 262).

**Data S1.** Transfusion‐transmitted malaria in India.

## Data Availability

The data that support the findings of this study are available from the corresponding author upon reasonable request.
