# Supplementary material for: Transfusion‐transmitted malaria in India: A national survey of epidemiology and testing practices
Source: Vox Sang. 2026 Mar 30;121(7):1010–9. doi: 10.1111/vox.70261 (PMC13356988; doi:10.1111/vox.70261)
Supplement: Supplementary file 1 — Table S1. Geographic distribution of included facilities in India (N = 262). Table S2. Facility‐reported predominant Plasmodium species among facilities self‐reporting location in malaria‐endemic areas, stratified by state/union territory. Table S3. Haemovigilance practices by region (N = 262). [file VOX-121-1010-s001.docx]

**Supplemental Table 1.** Geographic distribution of included facilities in India (N=262)

| **Broad region** | **State/union territory** | **Facilities, n** | **Percentage (%)** |
| --- | --- | --- | --- |
| North | Haryana | 61 | 23.3 |
| North | Punjab | 38 | 14.5 |
| North | Uttar Pradesh | 18 | 6.9 |
| North | Delhi | 14 | 5.3 |
| North | Rajasthan | 8 | 3.1 |
| North | Chandigarh | 4 | 1.5 |
| North | Himachal Pradesh | 2 | 0.8 |
| North | Uttarakhand | 1 | 0.4 |
| West | Gujarat | 26 | 9.9 |
| West | Maharashtra | 26 | 9.9 |
| South | Karnataka | 17 | 6.5 |
| South | Kerala | 10 | 3.8 |
| South | Tamil Nadu | 8 | 3.1 |
| South | Telangana | 5 | 1.9 |
| South | Andhra Pradesh | 2 | 0.8 |
| South | Puducherry | 1 | 0.4 |
| Central | Madhya Pradesh | 6 | 2.3 |
| Central | Chhattisgarh | 2 | 0.8 |
| East | West Bengal | 3 | 1.1 |
| East | Bihar | 1 | 0.4 |
| East | Odisha | 2 | 0.8 |
| Northeast | Assam | 5 | 1.9 |
| Northeast | Manipur | 1 | 0.4 |
| Northeast | Nagaland | 1 | 0.4 |
|  | **Total** | **262** | **100.0** |

***Note.*** *Percentages are based on the 262 included facilities. State/union territory assignment was based on the reported city of the responding institution. Broad regions were defined for descriptive purposes as North, West, South, Central, East, and Northeast India.*

**Supplemental Table 2.** Facility-reported predominant *Plasmodium* species among facilities self-reporting location in malaria-endemic areas, stratified by state/union territory

| **State/union territory** | **Endemic facilities, n** | ***P. vivax*, n** | ***P. falciparum*, n** | **Not sure, n** | **No response, n** |
| --- | --- | --- | --- | --- | --- |
| Haryana | 47 | 1 | 1 | 45 | 0 |
| Punjab | 27 | 0 | 0 | 27 | 0 |
| Gujarat | 15 | 10 | 3 | 2 | 0 |
| Maharashtra | 15 | 10 | 3 | 1 | 1 |
| Uttar Pradesh | 6 | 5 | 1 | 0 | 0 |
| Karnataka | 5 | 4 | 1 | 0 | 0 |
| Chhattisgarh | 2 | 1 | 0 | 1 | 0 |
| Himachal Pradesh | 1 | 0 | 0 | 1 | 0 |
| Delhi | 3 | 2 | 0 | 1 | 0 |
| Assam | 2 | 1 | 0 | 0 | 1 |
| Chandigarh | 2 | 1 | 1 | 0 | 0 |
| Tamil Nadu | 2 | 0 | 1 | 1 | 0 |
| West Bengal | 2 | 1 | 0 | 1 | 0 |
| Andhra Pradesh | 1 | 0 | 1 | 0 | 0 |
| Bihar | 1 | 0 | 1 | 0 | 0 |
| Kerala | 1 | 0 | 1 | 0 | 0 |
| Madhya Pradesh | 1 | 0 | 1 | 0 | 0 |
| Odisha | 1 | 1 | 0 | 0 | 0 |
| Rajasthan | 1 | 1 | 0 | 0 | 0 |
| Uttarakhand | 1 | 1 | 0 | 0 | 0 |
| **Total** | **136** | **39** | **15** | **80** | **2** |

***Note****. Among the 136 facilities that self-reported being in malaria-endemic areas, 134 answered the species question. State/union territory was assigned from the reported city/institution field for descriptive purposes.*

**Supplemental Table 3.** Hemovigilance practices by region (N = 262)

| **Region** | **N** | **Reports to hemovigilance program** | **Performs lookback investigations** | **Contacts prior recipients** |
| --- | --- | --- | --- | --- |
| North | 146 | 46 (32%) | 25 (17%) | 19 (13%) |
| South | 43 | 29 (67%) | 29 (67%) | 21 (49%) |
| West | 52 | 41 (79%) | 23 (44%) | 14 (27%) |
| East | 6 | 4 (67%) | 3 (50%) | 1 (17%) |
| Central | 8 | 5 (62%) | 4 (50%) | 4 (50%) |
| Northeast | 7 | 5 (71%) | 4 (57%) | 4 (57%) |
| Total | 262 | 130 (50%) | 88 (34%) | 63 (24%) |

***Note****. Data derived from survey of 262 blood collection facilities across India, 2025. Hemovigilance reporting, lookback investigations, and contact of prior recipients are reported as n (%) of facilities within each region.*
