# Supplementary material for: Transfusion‐transmitted malaria in India: A national survey of epidemiology and testing practices
Source: Vox Sang. 2026 Mar 30;121(7):1010–9. doi: 10.1111/vox.70261 (PMC13356988; doi:10.1111/vox.70261)
Supplement: Supplementary file 2 — Data S1. Transfusion‐transmitted malaria in India. [file VOX-121-1010-s002.pdf]

Transfusion-Transmitted Malaria in India

Dear colleague,

The objective of this survey is to assess practices for testing for transfusion-transmitted malaria in India. Your respones will help us to better understand the scope of available testing modalities and the epidemiology of transfusion-transmitted malaria. This survey asks for information which you may not have readily available at the time you initiate the survey. You are welcome to pause the survey as many times as needed to obtain the information, and then return to complete the survey, as your answers will be saved.

Your participation in this survey is voluntary and anonymous. Data will be aggregated and analyzed in a way that ensures individual responses cannot be identified. All responses will remain strictly confidential. By completing this survey, you consent to the use of your responses for research purposes while maintaining your privacy.

Please complete the survey below.

Thank you!

Consent to participate: No personal or identifying information will be shared. Do you consent to have de-identified data collected in this study shared for research purposes?

- ◯ Yes
- ◯ No

What is your designation/role at your institution (e.g., medical director, laboratory supervisor)?

____

What is the name of your institution?

____

What city is your institution located in?

____

How would you classify your organization?

- ◯ Public
- ◯ Private
- ◯ Governmental organization
- ◯ Non-governmental organization (NGO) / Charitable organization
- ◯ Other

If you selected "Other", please explain how you would classify your facility

____

In 2024, how many whole blood donations were performed at your institution?

- ◯ Less than 1,000
- ◯ 1,000-4,999
- ◯ 5,000-9,999
- ◯ 10,000-24,999
- ◯ 25,000-49,999
- ◯ 50,000-99,999
- ◯ 100,000 or more

In 2024, how many apheresis platelet donations were performed at your institution?

- ◯ 0-249
- ◯ 250-499
- ◯ 500-999
- ◯ 1,000-2,499
- ◯ 2,500-4,999
- ◯ 5,000-9,999
- ◯ 10,000-24,999
- ◯ 25,000-49,999
- ◯ 50,000-99,999
- ◯ 100,000 or more

In 2024, how many apheresis red blood cell donations were performed at your institution?

- ◯ 0-249
- ◯ 250-499
- ◯ 500-999
- ◯ 1,000-2,499
- ◯ 2,500-4,999
- ◯ 5,000-9,999
- ◯ 10,000-24,999
- ◯ 25,000-49,999
- ◯ 50,000-99,999
- ◯ 100,000 or more

Is your facility located in an area that is considered to be endemic for malaria?

- ◯ Yes
- ◯ No
- ◯ Not sure

What is the most common Plasmodium species in your region?

- ◯ P. falciparum
- ◯ P. vivax
- ◯ P. ovale
- ◯ P. malariae
- ◯ P. knowlesi
- ◯ Not sure

When a blood donor is deferred for 3 months because they tested positive for malaria, are they re-tested after the three month deferral period to determine if they can re-enter the blood donor pool?

- ◯ Yes (blood donors are re-tested for malaria to determine if they can re-enter the blood donor pool)
- ◯ No (blood donors are not re-tested for malaria after the three month deferral period and are automatically re-entered into the blood donor pool)
- ◯ Not sure

Do you perform nucleic acid testing for any of the following? (Select all that apply)

- ☐ Human immunodeficiency virus (HIV)
- ☐ Hepatitis B virus (HBV)
- ☐ Hepatitis C virus (HCV)
- ☐ Malaria
- ☐ None
- ☐ Other (please specify)

Please list other agents that you perform nucleic acid testing for

____

Do you routinely test all units for malaria?

- ◯ Yes
- ◯ No

How do you test for malaria? (Select all that apply)

- ☐ Light microscopy of peripheral blood smear
- ☐ Antigen testing by rapid/point-of-care test
- ☐ Antigen testing by formal laboratory test (ie, automated/semi-automated assays)
- ☐ Molecular testing (nucleic acid amplification technology)
- ☐ Antibody tests

In the case of a positive test, do you identify the malaria species?

- ◯ Yes
- ◯ No
- ◯ Only to differentiate P. falciparum vs non-P. falciparum

Please provide the approximate percentage of positive tests by species

Species

Percentage of positive tests
P. falciparum ______
P. vivax ______
P. ovale ______
P. malariae ______
P. knowlesi ______

If the malaria screening test is reactive (i.e., positive), do you repeat the test using the same test method (i.e., repeat testing)?

- ◯ Yes
- ◯ No

If the malaria screening test is reactive (i.e., positive), do you repeat the test using a different test method (i.e., confirmatory testing)?

- ◯ Yes
- ◯ No

Are there external quality controls for verification of tests?

- ◯ Yes
- ◯ No
- ◯ Not sure

Do you use anti-malarial therapy in patients prior to transfusion?

- ◯ Yes
- ◯ No
- ◯ Not sure

In the event of unexplained fever following transfusion is routine testing for malaria undertaken?

- ◯ Yes
- ◯ No
- ◯ Not sure

Do you perform lookback investigations?

- ◯ Yes
- ◯ No

Do you contact recipients of prior donations?

- ◯ Yes
- ◯ No

In the event of lookback, how far back from the index donation is investigated?

- ◯ Up to 3 months
- ◯ >3 months to 6 months
- ◯ >6 months to 12 months
- ◯ Up to 2 years
- ◯ Anytime
- ◯ Not sure

Who oversees donor notification and management for your facility?	◯ Physician at blood center<br>◯ Blood bank counselor / medical social worker<br>◯ Physician in community or at a hospital<br>◯ Public health department<br>◯ Other<br>◯ Not sure
Please specify who oversees donor notification and management for your facility	__________________________________
Have you encountered cases of transfusion-transmitted malaria at your facility in the last 5 years (2020-2024)?	◯ Yes<br>◯ No<br>◯ Not sure
What blood product(s) were implicated in transfusion-transmitted malaria? (Select all that apply)	☐ Whole blood<br>☐ Red blood cells<br>☐ Platelets
How many transfusion-transmitted malaria cases have you encountered at your facility in the last 5 years (2020-2024)?	____________________________
Which of the following populations have you encountered transfusion-transmitted malaria in? (Select all that apply)	☐ Pregnant patients<br>☐ Children<br>☐ Immunocompromised<br>☐ Other
What other patient populations have you encountered transfusion-transmitted malaria in?	__________________________________
Do you report transfusion-transmitted malaria cases to a hemovigilance program?	◯ Yes<br>◯ No
If you have encountered cases of transfusion-transmitted malaria with whole blood or red blood cell transfusion, how long was the blood product stored for? (Select all that apply)	◯ Less than 7 days<br>◯ 7 to 14 days<br>◯ 15 to 21 days<br>◯ More than 21 days<br>◯ Not sure
Do you think that donor screening for malaria should be discontinued?	◯ Yes<br>◯ No<br>◯ Not sure<br>◯ Prefer not to answer

Over the last 5 years, approximately how many total units (whole blood and apheresis) were collected and how many units were positive for malaria?

	Total units collected	Units positive for malaria
2020	______	______
2021	______	______
2022	______	______
2023	______	______
2024	______	______
